# Overexpression of Rat Neurons Nitric Oxide Synthase in Rice Enhances Drought and Salt Tolerance

**DOI:** 10.1371/journal.pone.0131599

**Published:** 2015-06-29

**Authors:** Wei Cai, Wen Liu, Wen-Shu Wang, Zheng-Wei Fu, Tong-Tong Han, Ying-Tang Lu

**Affiliations:** State Key Laboratory of Hybrid Rice, College of Life Sciences, Wuhan University, Wuhan, 430072, China; University of Minho, PORTUGAL

## Abstract

Nitric oxide (NO) has been shown to play an important role in the plant response to biotic and abiotic stresses in Arabidopsis mutants with lower or higher levels of endogenous NO. The exogenous application of NO donors or scavengers has also suggested an important role for NO in plant defense against environmental stress. In this study, rice plants under drought and high salinity conditions showed increased nitric oxide synthase (NOS) activity and NO levels. Overexpression of rat neuronal NO synthase (*nNOS*) in rice increased both NOS activity and NO accumulation, resulting in improved tolerance of the transgenic plants to both drought and salt stresses. *nNOS*-overexpressing plants exhibited stronger water-holding capability, higher proline accumulation, less lipid peroxidation and reduced electrolyte leakage under drought and salt conditions than wild rice. Moreover, *nNOS*-overexpressing plants accumulated less H_2_O_2_, due to the observed up-regulation of *OsCATA*, *OsCATB* and *OsPOX1*. In agreement, the activities of CAT and POX were higher in transgenic rice than wild type. Additionally, the expression of six tested stress-responsive genes including *OsDREB2A*, *OsDREB2B*, *OsSNAC1*, *OsSNAC2*, *OsLEA3* and *OsRD29A*, in *nNOS*-overexpressing plants was higher than that in the wild type under drought and high salinity conditions. Taken together, our results suggest that *nNOS* overexpression suppresses the stress-enhanced electrolyte leakage, lipid peroxidation and H_2_O_2_ accumulation, and promotes proline accumulation and the expression of stress-responsive genes under stress conditions, thereby promoting increased tolerance to drought and salt stresses.

## Introduction

Abiotic environmental factors, such as drought and high salinity, are significant plant stressors that greatly impact on plant development and productivity, leading to serious losses in yield. Rice is the most important food crop in Asia. Thus, in an era of rapid population growth and environmental problems, improving drought and salt tolerance of rice through biotechnology, besides its scientific interest, might have an important applied relevance.

Plants have developed a series of strategies to cope with drought and salt stresses, including regulating the expression of stress-responsive genes, scavenging ROS, accumulating proline, inducing stomatal closure, and maintaining low Na^+^ concentration in the cytosol by controlling Na^+^ efflux across the PM and tonoplast [1]. Moreover, plant hormones including abscisic acid (ABA), gibberellin, auxin, jasmonic acid and NO, also play important roles in stress adaptive signaling [1–4].

NO functions as a signaling molecule involved in a range of plant growth and developmental processes, including seed germination [5], root growth [6], floral regulation [7], plant maturation and senescence [8], as well as stomatal closure [9]. NO also participates in the plant response to various biotic and abiotic stresses, such as cold, drought, salt, heat and heavy metal stresses, and pathogen infection [10–14]. The various roles of NO in plant development and environmental adaptation suggest that the genetic manipulation to increase NO production may improve plant tolerance against adverse environmental conditions.

As a bioactive molecule, NO functions always depend on its location and concentration, as well as the species and developmental stages of plant. Thus, in different plant species, NO may play different roles in the same physiological processes. For example, NO acts as a positive mediator in Cd^2+^-induced ROS accumulation in yellow lupine and Arabidopsis suspension culture [15,16], but mediates apposite effects in *Brassica juncca* and rice seedlings [13,17]. Additionally, NO reduces aluminum toxicity in roots of *Cassia tora* L [18], but showed a synergistic effect on the Al^3+^-induced inhibition of root elongation in rice bean (*Vigna umbellate*) [19]. Therefore, exploring the roles of NO in rice is of great interest and importance.

Our knowledge about the roles of NO in plants has been achieved by exogenous application of NO donors or scavengers [11–13,17]. However, it is still not clear whether the observed changes of phenotypes that resulted from the application of the pharmacological compounds reflect the true physiological effects of NO, without side effects. For example, the treatment with three different NO donors, sodium nitroprusside (SNP), S-nitroso-N-acetyl-D-penicillamine (SNAP) and nitrosoglutathione (GSNO), showed different effects in several studies [20,21]. Therefore, it is suggested that in order to assess the involvement of NO in development and stress signaling, plant materials with endogenously higher or lower NO content should be used [22,23].

Although a series of experiments indicate the activity of arginine-dependent NO synthase (NOS) in higher plants, a *NOS* gene has not yet been found. While several mutants of the dicot Arabidopsis, including *nia1nia2*, *noa1* and *nox1*, showed altered NO levels [7,24,25], the mutant *noe1* of the monocot rice showed higher NO accumulation [26]. However, most of these genes do not directly participate in NO synthesis. For instance, *OsNOE1* encodes a rice catalase OsCATC, thus the observed NO accumulation in *Osnoe1* mutant could result from an increase of H_2_O_2_ [26].

To reveal the functional role of NO in stress response in rice, we drove overexpression of the rat neuronal *NOS* (*nNOS*) under the control of a ubiquitin promoter in rice (*Oryza sativa*) ZH11 and assayed the responses of these transgenic lines upon exposure to environmental stresses. Our results indicated that the *nNOS* transgenic plants with higher NO accumulation exhibited enhanced tolerance to both drought and salt stresses. Further analyses showed that the transgenic rice plants had stronger ROS-scavenging capacity, higher proline accumulation, stronger water-holding capability and increased expression of stress-responsive genes under such stress conditions.

## Material and Methods

### Ethics statement

The full-length cDNA fragment of rat *nNOS* was obtained from the nNOSPCW plasmid, which was provided by Professor Bettie Sue Siler Masters [22]. We did not use any animals in our experiments.

### Plant materials and growth conditions

Rice (*Oryza sativa* L. cv. Zhonghua11) was used to generate transgenic plants. Rice seeds were sterilized in 5% NaClO for 30 min, and thoroughly rinsed with distilled de-ironed water. The seeds were germinated and cultured in 1/2 MS (Murashige and Skoog) media (50% humidity, 200 μmol m^-2^s^-1^, 16h light/8h dark cycle, 28°C–30°C). Seven-day-old plants were then transferred from 1/2 MS media to soil in the greenhouse (50% humidity, 400 μmol m^-2^s^-1^, 16h light/8h dark cycle, 28°C–30°C).

### Stress treatments and plant sampling

To evaluate the plant tolerance to NaCl or mannitol stress, 3-day-old seedlings in 1/2 MS media were transferred to 1/2 MS media supplemented with 200 mM mannitol or 200 mM NaCl. After 10 days, seedlings were photographed and shoot length, fresh weight and relative water content were measured.

To assay drought stress tolerance of the transgenic plants in soil, six 7-day-old plants from each line were grown on 1/2 MS media and were then transplanted into 12 L plastic pots (30 cm in diameter and 25 cm in depth) filled with 7.5 kg paddy soil, which was plowed and harrowed 3 days before planting, in the greenhouse (50% humidity, 400 μmol m^-2^s^-1^, 16h light/8h dark cycle, 28°C–30°C) and grown for an additional 4 weeks. The pH (H_2_O) of the topsoil (0–15 cm) was 6.5. Then, both wild-type and transgenic plants grown in the soil were subjected to drought treatment by pouring out all surface water and stopping irrigation for 2 weeks. The surviving seedlings were photographed and analyzed after re-watering for 7 days. After drought treatment for 2 weeks, the treated seedlings became wilted. The leaves of surviving seedlings turned green after recovery in 7 days, while the leaves of the dead seedlings were completely wilted and pale.

To measure the physiological parameters and the transcript levels of the related genes, including MDA content, electrolyte leakage, H_2_O_2_ content, the activities of CAT and POX, the expression of stress responsive genes and *nNOS*, NOS activity, NO fluorescence and content, the second leaves of 16-day-old plants were used. Plants grown in 1/2 MS media were used as control. For stress treatments, the roots of 2-week-old (3-leaf stage) wild-type and transgenic plants grown on 1/2 MS media were submerged in either water, 200 mM mannitol or 200 mM NaCl solution for designated times and then used to measure NO content and NOS activity. Plants submerged for two days were sampled for other parameters. The flag leaves of plants grown in soil were used to measure water loss rate and stomatal conductance.

### Characterization of transgenic lines overexpressing rat *nNOS* in rice

To make the construct for *nNOS* overexpression in rice, the full-length cDNA fragment of *nNOS* from the nNOSPCW plasmid [27] was inserted into the pUbiO vector. The construct was then introduced into the *Agrobacterium tumefaciens* strain EHA105 for *Agrobacterium*-mediated transformation of *japonica* rice Zhonghua11 [28]. The transgenic lines were selected on the basis of hygromycin resistance and genomic PCR. The homozygous T3 generation of transgenic rice plants was used in subsequent stress experiments.

### Mesurement of endogenous NO content

Endogenous NO levels were determined by using the NO-specific fluorescent probe DAF-FM DA, as described in previous reports [22,26,29]. For staining of DAF-FM DA, 2 cm segments were excised from the second leaves of transgenic and control rice seedlings, which were grown in 1/2 MS media. The segments were then incubated in a 2 mL EP tube with 1.8 mL of buffer (10 μM DAF-FM DA, 20 mM HEPES-NaOH, pH 7.5) for 1 h, and rinsed three times with distilled water for 5 min to remove excess probe. For imaging, the samples were mounted in glycerol: distilled water (1:1 v/v) in preparation for examination with an Olympus BX60 differential interference contrast (DIC) microscope, equipped with a Charge-Coupled Device (CCD) Olympus dp72. The excitation wavelength was 488 nm and the emission wavelength was 515 nm. The signal intensity was measured using Image J software (http://rsb.info.nib.gov/ij/). 20 to 30 leaves of each line were observed per experiment.

In order to determine the NO content, a previously described method using an NO-selective electrode was also employed in our study [7]. About 0.5 g of rice leaves were ground with liquid nitrogen, resuspended in the buffer (0.1 mM CaCl_2_, 10 mM KCl, 10 mM MES-Tris, pH 5.6) and used for the measurement of NO content with an ISO-NO Mark II NO meter (World Precision Instruments). The standard calibration curve of NO was generated using the aqueous standards, prepared by chemically generating NO. The NO concentration was determined with the Duo 18 data acquisition system (World Precision Instruments).

### Measurement of NOS activity

NOS activity was measured as previously described [22]. Briefly, about 0.5 g of rice leaves were frozen and ground with liquid nitrogen, and extracted with 2 mL buffer (50 mM Tris–HCl, pH 7.4, 1 mM EDTA, 1 mM dithiothreitol, 1 mM leupeptin, 1 mM pepstatin, and 1 mM phenylmethylsulfonyl fluoride). After centrifuging at 12,000 g for 15 min at 4°C, the supernatant was used as the enzyme extract. NOS activity was assayed using a NOS assay kit based on DAF-FM DA [30].

### Measurement of relative water content

Relative water content (RWC) was measured according to a previously described method [31]. To determine relative water content (RWC), 20 leaves (the second leaf) from plants grown on 1/2 MS media with or without 200 mM NaCl or 200 mM mannitol were detached and weighted to obtain the fresh weight (FW). Then, these leaves were soaked in de-ionized water for 4 h and saturated weight (SW) was measured. The leaves were dried for 48 h at 80°C to determine dry weigh (DW). RWC were calculated as follows: RWC = (FW - DW)/(SW - DW) × 100%.

### Measurement of proline content and water loss rate

Proline content in rice leaves was measured according to a previously described method [22]. About 0.5 g of rice leaves were ground into powder with liquid nitrogen and extracted in 3% sulfosalicylic acid. After centrifuging at 12,000 g for 10 min, the supernatant (2 mL) was mixed with 2 mL of ninhydrin reagent [2.5% (w/v) ninhydrin, 60% (v/v) glacial acetic acid and 40% 6 M phosphoric acid] and 2 mL of glacial acetic acid, incubated at 100°C for 40 min. Then, the reaction was terminated in an ice bath. The reaction mixture was extracted with 4 mL of toluene and the absorbance was measured at 520 nm with a UV-5200 spectrophotometer.

To measure the water loss rate of the detached leaves from the wild type and transgenic plants, 6 flag leaves were detached and placed onto clean filter paper at room temperature. The fresh weight loss was measured during the designated time points.

### Measuerment of stomatal conductance

Stomatal conductance was measured as previously described [32]. Stomatal conductance (gs, mol H_2_O m^-2^s^-1^) was determined (LI-6400XT, LI-COR Inc, Lincoln, NE, USA) on the flag leaf. LI-COR leaf chamber conditions were controlled at 400 ppm CO_2_, flow rate (300 μmol s^-^), 28°C (block temperature), PAR (1000 μmol m^-2^s^-1^) and 50% relative humidity. Twelve plants per line were used with three independent biological replicates.

### Measurement of electrolyte leakage

The electrolyte leakage was measured according to the method described previously [26, 33]. Six detached leaves, from either the wild type or transgenic lines, were placed into a 100-mL beaker containing 40 mL of distilled de-ionized water, shaken at 120 rpm for 3 h and used to measure conductivity (C1) with an ion leakage meter. Then, the leaves were boiled for 30 min and shaken for 1 h and used for conductivity (C2). The electrolyte leakage was calculated as follows: (C1/C2) × 100%.

### Measurement of MDA content

MDA content was measured according to the method described previously [22]. Briefly, about 0.5 g of rice leaves were ground in 2 mL of the chilled reagent [0.25% (w/v) thiobarbituric acid in 10% (w/v) trichloroacetic acid]. The extracts were incubated at 100°C for 30 min, cooled to room temperature and centrifuged at 12,000 g for 15 min. The absorbance of the supernatant was measured at 450, 532 and 600 nm. The MDA content was calculated based on the following equation: 6.45× (OD_532_-OD_600_)-0.559×OD_450_.

### Quantification of H_2_O_2_

Hydrogen peroxide (H_2_O_2_) content was measured by peroxidase-coupled assay protocols as described previously [34,35]. This assay is based on absorbance changes at 590 nm.

### Measurement of POX activity and CAT activity

To measure POX and CAT activity, total protein from rice leaves was extracted with 0.05 M potassium phosphate buffer (pH 7.0). After centrifuging at 12,000 g for 15 min at 4°C, the supernatant was used for the measurement of POX and CAT activities. Peroxidase activity was determined using the previously described method [36]. The five mL reaction mixture contained 0.1 mL of the supernatant, 2.9 mL of 0.05 M potassium phosphate buffer (pH 5.5), 1 mL of 0.5% (v/v) H_2_O_2_ and 1 mL of 0.05 M guaiacol as substrates. The oxidation of guaiacol was monitored by the absorbance measured at 470 nm every 10 s. Catalase activity was confirmed using a Catalase Assay Kit (Beyotime) according to the manufacturer’s instructions.

### Semi-quantitative RT–PCR and quantitative real-time PCR

Total RNA was extracted from rice leaves using TRIzol reagent (Invitrogen). 1μg of total RNA treated with RQ1 RNase-free DNase (Promega) was used to synthesize cDNA with an RT kit (TOYOBO) according to the manufacturer’s instructions. The quantitative RT-PCR assay was performed on a Bio-Rad CFX96 apparatus with the dye SYBR Green I (Invitrogen). PCR was carried out in 96-well plates with the following settings: 3 min incubation step at 95°C for complete denaturation, followed by 50 cycles consisting of 95°C for 15 s and 60°C for 30 s. The rice gene *eEF1α* was chosen as the reference gene for the following analysis according to geNorm software (http://medgen.ugent.be/~jvdesomp/genorm/). All experiments were performed with three independent biological replicates and three technical replicates. Gene-specific primers for qRT-PCR in this study are listed in Supporting Information S1 Table.

### Statistical analysis

All experiments in this study were repeated at least three times. The *t*-test was used to determine the statistical significance of the results. Asterisk symbols (*) indicate significant differences: * (*P* < 0.05), ** (*P* < 0.01) and *** (*P* < 0.001).

## Results

### Overexpressing rat *nNOS* in rice increases both NOS activity and NO content

**Fig 1:**
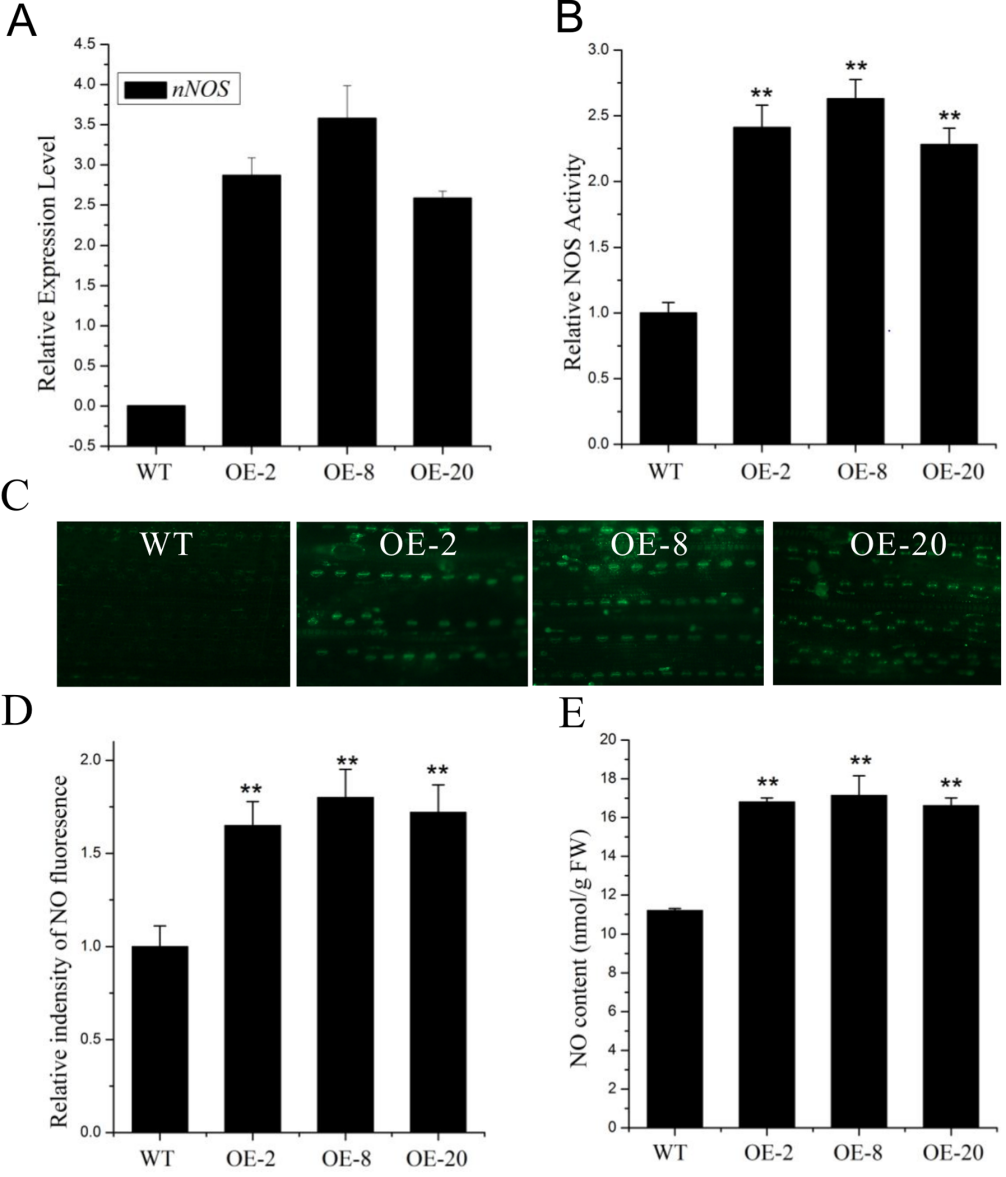
Identification and characterization of *nNOS-*overexpressing lines. (A) *nNOS* expression in the selected transgenic lines. The mRNA levels of *nNOS* in *nNOS*-overexpressing lines and the wild type were examined by quantitative RT–PCR analyses with *eEF1α* expression as the internal control. (B) NOS activities of the *nNOS*-overexpressing lines and wild-type plants were determined using a NOS assay kit. The relative NOS activity was expressed using the NOS activity of the wild type as the standard (1). (C and D) NO fluorescence in leaves of the *nNOS*-overexpressing lines and wild-type plants examined using DAF-FM DA (C) and the relative NO content (D) expressed using the fluorescence of the wild type as the standard (1). (E) NO contents of the leaves of both the *nNOS*-overexpressing lines and the wild type examined using an electrode-based method described in the Materials and Methods. The results shown are the mean ± SD. Values are derived from three independent biological experiments. ** (*P* < 0.01) indicates significant differences in comparison with wild-type control respectively (Student’s *t*-test).

To modulate NO content in rice, we overexpressed the *nNOS* gene in *japonica* rice Zhonghua11 (ZH11) by inserting the coding region of the rat *nNOS* [22] into the pUbiO plant expression vector, which was then introduced into ZH11 via *Agrobacterium*-mediated transformation [28]. Following selection on 1/2 MS media containing 50 mg/L hygromycin, the independent transgenic plants were transferred to soil in the greenhouse. Genomic PCR analysis was used to verify the *nNOS* insertion and NOS activity was measured in each line. Three transgenic lines, #2, #8 and #20 (OE-2, OE-8 and OE-20), were used for further experiments. Our quantitative RT-PCR data indicated that the selected lines highly expressed *nNOS* (Fig 1A) and showed that NOS activity was 2.41, 2.63 and 2.28 times higher than wild type, respectively (Fig 1B).

Then, we further examined whether the increased NOS activity in these three transgenic lines resulted in altered NO content by staining with the NO-sensitive dye, 3-amino, 4-aminomethyl-2’, 7’-difluorescein diacetate (DAF-FM DA). Our results showed that all three lines had much higher levels of NO compared with the wild type (Fig 1C and 1D). The NO content of these three lines was also verified using a method based on an NO-selective electrode (Fig 1E). Taken together, our data indicate that *nNOS* overexpression can increase NOS activity, leading to NO accumulation in the transgenic rice lines.

### *nNOS* overexpression confers tolerance of transgenic lines to both drought and salt stresses

**Fig 2:**
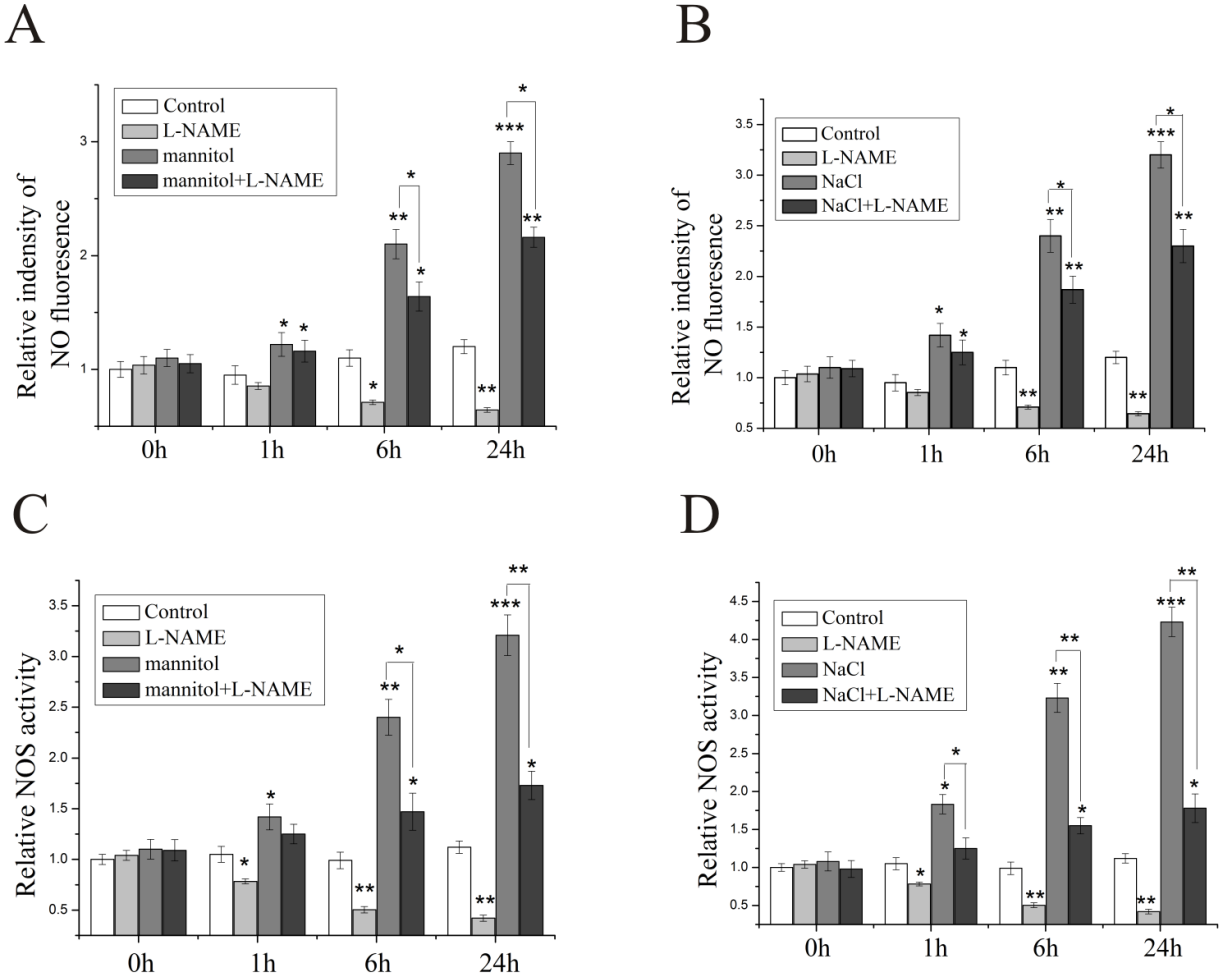
Endogenous NO level and NOS activity in rice leaves subjected to mannitol and NaCl stresses. (A and B) Effect of mannitol and NaCl stresses on the endogenous NO level in the leaves of wild-type rice plants. Two-week-old wild-type plants were treated with either 200 mM mannitol or 200 mM NaCl combined with or without 500 μM L-NAME for 0, 1, 6 and 24 h, and then NO production was measured using DAF-FM DA. The relative NO content (A and B) was expressed using the fluorescence of the control at 0 h as the standard (1). (C and D) The NOS activity was determined using a NOS assay kit. The relative NOS activity (C and D) was expressed using the NOS activity of the control at 0 h as the standard (1). The results shown are the mean ± SD. Values are derived from three independent biological experiments. * (*P* < 0.05), ** (*P* < 0.01) and *** (*P* < 0.001) indicate significant differences in comparison with the water-treated control respectively (Student’s *t*-test).

NO has been shown to play an important role in plant responses to many different abiotic stresses by using NO donors or scavengers [37]. However, knowledge of how endogenous NO functions in plant responses to these stresses is still limited. Drought and salt are major stress factors that limit agricultural production worldwide. Thus, we examined possible changes of NOS activity and NO content in rice subjected to both drought and salt stresses. For this purpose, the roots of 2-week-old wild-type rice seedlings (3-leaf stage) were submerged into 200 mM mannitol, which mimicked drought stress as previously reported [36,38,39], and both NOS activity and NO content were assayed at 0, 1, 6 and 24 hours post treatment. Our data revealed that mannitol treatment induced NO accumulation in rice leaves (Fig 2A), which was similar to a previous study that reported a drought-mediated increase in NO levels [29]. When rice plants were treated with 200 mM NaCl, NO content was increased to 3.21 times 24 h after treatment (Fig 2B). The increase in NO levels of rice leaves subjected to either mannitol or NaCl stress could be a result of changes in NOS activity. As expected, NOS activity was increased to 3.18 times in response to mannitol and 4.23 times in response to NaCl (Fig 2C and 2D). To further confirm it, the NOS inhibitor, L-N^G^-nitro arginine methylester (L-NAME) was used in subsequent experiments. Our results showed that treatment with L-NAME repressed both mannitol and NaCl-mediated increase in NOS activity, resulting in a lower NO concentration (Fig 2), suggesting that both drought and salt stresses can modulate NO content, perhaps partially through NOS activity.

**Fig 3:**
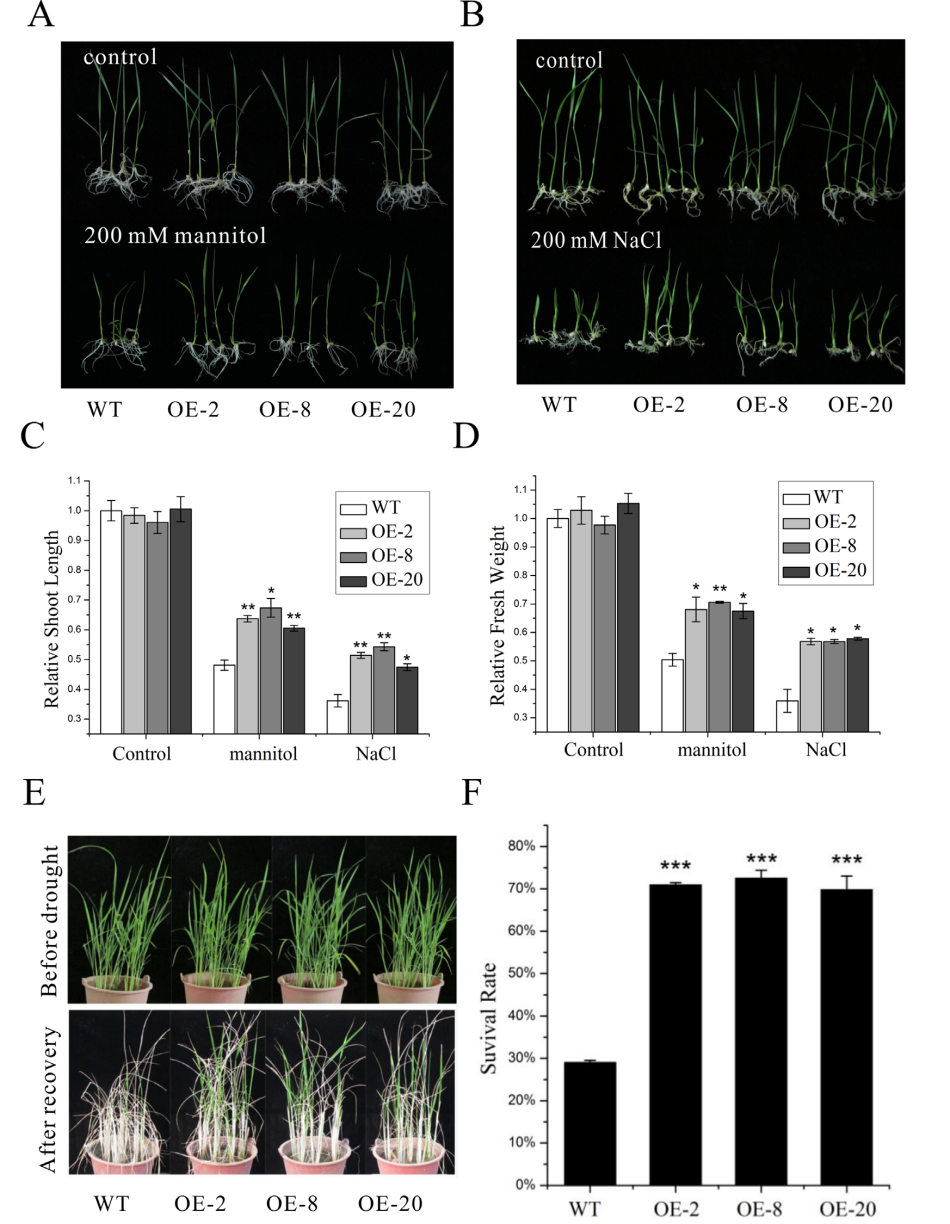
The *nNOS-*overexpressing lines show enhanced drought and salt tolerance. (A and B) The appearance of 200 mM mannitol (A) or 200 mM NaCl (B) treated wild-type plants and *nNOS*-overexpressing lines at 10 d after germination. (C and D) Relative shoot length (C) and fresh weight (D) were assayed with both wild-type plants and three *nNOS*-overexpressing lines at 10 day after growth on the media (1/2 MS with or without 200 mM mannitol or 200 mM NaCl). The results shown are the mean ± SD. Values are derived from three independent biological experiments. (E and F) The appearance (E) and survival rates (F) of five-week-old wild-type and *nNOS*-overexpressing plants subjected to drought stress for 2 weeks and followed by re-watering for 7 d. The results shown are the mean ± SE (n = 3). * (*P* < 0.05), ** (*P* < 0.01) and *** (*P* < 0.001) indicate significant differences in comparison with wild-type control respectively (Student’s *t*-test).

Then, the tolerance of transgenic rice plants to drought and salt stresses was investigated. For this purpose, the seedlings of both wild-type and transgenic lines were transferred onto 1/2 MS media supplemented with either 200 mM NaCl or 200 mM mannitol, and both shoot length and fresh weight were assayed 10 days following transfer. All tested transgenic lines exhibited similar shoot length and fresh weight to the wild type in normal 1/2 MS media. However, the transgenic lines were less sensitive to both mannitol and NaCl stresses, exhibiting a smaller decrease in both shoot length and fresh weight than wild type (Fig 3A–3D), indicating that *nNOS* overexpression in rice can improve drought and salt tolerance at the seedling stage.

Plant tolerance to drought stress was also tested using plants grown in soil. Under normal growth conditions, the transgenic plants did not display any obvious difference compared to wild type. Then, five-week-old plants were subjected to drought treatment by stopping irrigation for 2 weeks. The leaves of these transgenic lines were less wilted compared to those of the wild type (Fig 3E). Finally, the survival rates of wild-type and transgenic plants were statistically analyzed one week after recovery. Our data showed that approximately 69% to 72% of the *nNOS*-overexpressing plants survived, whereas only 29% of wild-type plants survived after recovery (Fig 3E and 3F), further indicating that overexpressing the rat *nNOS* can significantly improve drought tolerance in rice.

### The transgenic plants modify the changes in physiological parameters under drought and salt stresses

It is well known that both drought and salt stresses cause numerous changes in the physiology and metabolism of plants. Our transgenic rice plants may modify the stress-mediated changes of these physiological parameters, resulting in higher tolerance to both drought and salt stresses. Thus, several physiological parameters including relative water content (RWC), water loss rate, stomatal conductance, proline content, electrolyte leakage and malondialdehyde (MDA) content, were examined in these transgenic lines.

**Fig 4:**
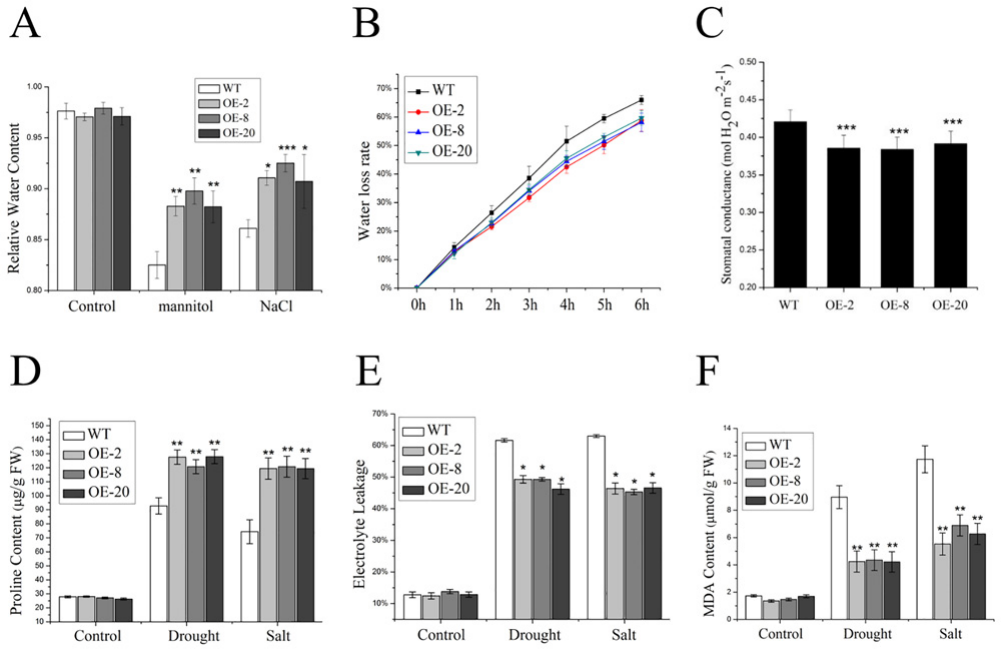
Changes of physiological parameters of both *nNOS*-overexpressing lines and wild-type plants under drought and salt stresses. (A) Relative water content of wild-type and transgenic lines under mannitol and NaCl stresses. Relative water content (RWC) was assayed with both wild-type plants and *nNOS*-overexpressing lines at 10 day after growth on the media (1/2 MS with or without 200 mM mannitol or 200 mM NaCl). The results shown are the mean ± SD. (B) Water loss rate from the detached leaves of both wild-type plants and *nNOS*-overexpressing lines at indicated time points. The results shown are the mean ± SD. (C) Stomatal conductance was assayed on the flag leaves of both wild-type plants and *nNOS*-overexpressing lines. The results shown are the mean ± SD. (D-F) Two-week-old wild-type and *nNOS*-overexpressing plants were treated with either 200 mM mannitol or 200 mM NaCl for 2 days with water treatment as control, and then proline content (D), electrolyte leakage (E) and MDA content (F) were assayed. The results shown are the mean ± SE (n = 3). * (*P* < 0.05), ** (*P* < 0.01) and *** (*P* < 0.001) indicate significant differences in comparison with wild-type control respectively (Student’s *t*-test).

RWC, water loss rate and stomatal conductance of the leaf reflect water-holding capability of plants. Thus, the RWC of rice leaves was measured. When subjected to either salt or mannitol stress, whereas the RWC of both wild-type and transgenic lines was reduced compared with untreated controls, the RWC of transgenic plants was significantly higher than that of the wild type (Fig 4A). The differences between the wild type and transgenic lines could be attributed to the different water loss rates of these plants. Indeed, the detached leaves of wild-type plants lost water faster than those of the transgenic lines (Fig 4B). In addition, lower stomatal conductance was observed in the transgenic lines compared with that of the wild type (Fig 4C). These results suggest that the transgenic lines have stronger water-holding capability.

Proline accumulation is considered to be an adaptive response of plants against environmental stresses such as heavy metal, high salinity and drought [29]. Under normal growth conditions, the proline contents in our transgenic lines with higher NO accumulation were similar to that of wild-type plants. When subjected to either drought or salt stress, both the wild type and transgenic lines increased proline contents. However, all three transgenic lines showed significantly higher proline accumulation than the wild type (Fig 4D).

The electrolyte leakage and MDA content are important indexes of cell damage in the plant stress response [22,36]. Our data indicated that both wild-type and transgenic rice plants had similar electrolyte leakage under normal growth conditions, but the electrolyte leakage of wild-type plants was much higher than that of transgenic lines after drought and salt treatment (Fig 4E). The content of MDA as a by-product of the oxidation of polyunsaturated fatty acids reflects the level of lipid peroxidation. Similar to electrolyte leakage, the MDA content of the transgenic lines was much lower than that of the wild type under drought and salt stresses (Fig 4F). Taken together, the enhanced drought and salt tolerance of the *nNOS* transgenic plants may be due to stronger water-holding capability, improved proline accumulation, less electrolyte leakage and MDA content under stress conditions.

### The *nNOS*-overexpressing rice plants enhance ROS- scavenging capacity

**Fig 5:**
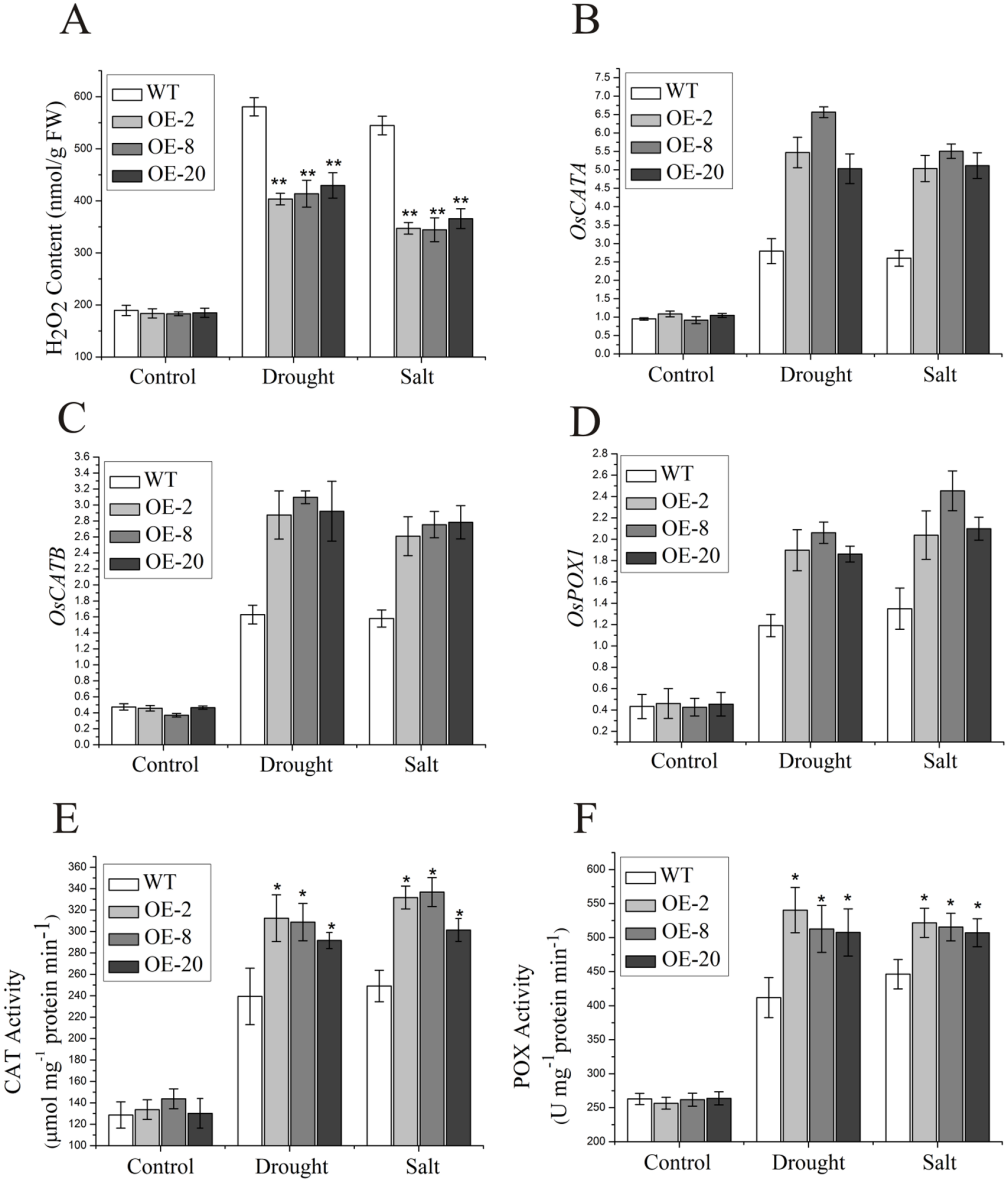
The *nNOS-*overexpressing lines exhibit improved ROS-scavenging capacity under drought and salt stresses. Two-week-old wild-type and *nNOS*-overexpressing plants were treated with either 200 mM mannitol or 200 mM NaCl for 2 days with water treatment as control, and then H_2_O_2_ contents (A), the expression of *OsCATA* (B), *OsCATB* (C) and *OsPOX1* (D) and enzymatic activities of CAT (E) and POX (F) were assayed. The results shown are the mean ± SE (n = 3). * (*P* < 0.05) and ** (*P* < 0.01) indicate significant differences in comparison with wild-type control respectively (Student’s *t*-test).

It is reported that much of the drought/salt-caused injury at the cellular level is associated with oxidative damage due to ROS [1]. The lower level of lipid peroxidation, as shown by the lower MDA content in the transgenic plants compared with that of wild type, may be associated with reduced ROS accumulation under drought and salt stresses. It is also known that treatment with SNP, an NO donor, can alleviate the oxidative damage in drought/salt-stressed marigold, cucumber and wheat [11,12,40,41]. Therefore, we expected that our transgenic plants with higher NO content might also repress drought/salt-induced ROS accumulation, leading to higher tolerance to the stresses. For this purpose, we assayed H_2_O_2_ content of both wild-type and transgenic plants. Indeed, the transgenic plants showed less drought and salt stress-induced H_2_O_2_ accumulation compared to wild type, while both wild-type and transgenic plants had similar levels of H_2_O_2_ under normal conditions (Fig 5A). This suppression of stress-induced H_2_O_2_ accumulation in the transgenic lines could result from changes in expression of antioxidant enzyme genes such as *OsCATA*, *OsCATB* and *OsPOX1*. As expected, our qRT-PCR analyses for expression levels of these genes indicated that the transgenic lines highly expressed these genes compared to the wild type, when subjected to both drought and salt stresses (Fig 5B–5D). Consistent with this, compared to the wild type, the transgenic plants had much higher CAT and POX activities under drought and high salinity conditions, but similar activities under normal conditions (Fig 5E and 5F). These results suggest that the *nNOS*-overexpressing rice plants have enhanced ROS-scavenging capacity by increasing the expression of antioxidant enzyme genes under stress conditions.

### The transgenic rice plants change the expression of stress-responsive genes under drought and high salinity conditions

**Fig 6:**
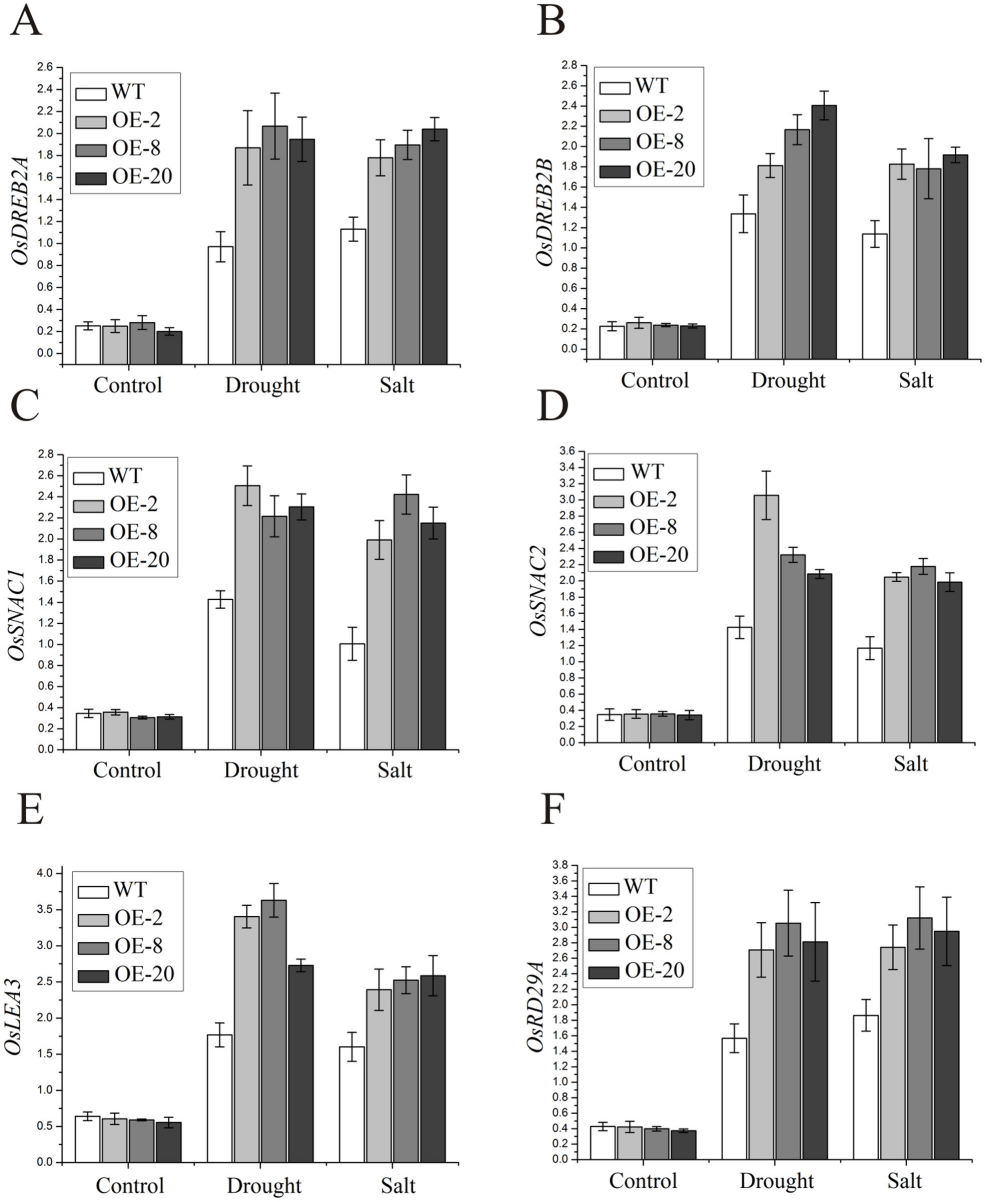
The expression of stress-responsive genes in *nNOS-*overexpressing lines subjected to drought and salt stresses. (A-F) The expression of *OsDREB2A* (A), *OsDREB2B* (B), *OsSNAC1* (C), *OsSNAC2* (D), *OsLEA3* (E) and *OsRD29A* (F) in wild-type plants and *nNOS*-overexpressing lines under normal, drought and salt conditions was assayed by quantitative real-time PCR. *eEF1α* was used to normalize expression of these genes. The results shown are the mean ± SE (n = 3).

When challenged with either drought or salt stress, the plant up-regulates the expression of stress-responsive genes, including *OsDREB2A*, *OsDREB2B*, *OsSNAC1*, *OsSNAC2*, *OsLEA3* and *OsRD29A*. Overexpression of these genes leads to enhanced tolerance to abiotic stresses [42]. Thus, we assayed the expression levels of *OsDREB2A*, *OsDREB2B*, *OsSNAC1*, *OsSNAC2*, *OsLEA3* and *OsRD29A* by qRT-PCR. Both wild-type and transgenic lines exhibited similar expression levels of all the tested genes under normal conditions (Fig 6). However, when subjected to either drought or salt stress, the expression levels of all of these genes were significantly higher in the transgenic lines compared with those in the wild type (Fig 6), suggesting that the transgenic lines with higher NO content are more tolerant to the stresses, possibly by highly stimulating the expression of stress-responsive genes.

## Discussion

In the last two decades, rapidly increasing evidence has indicated that NO is a key player in the plant response to many different stresses. However, there has been much disagreement regarding the mechanism by which NO acts in these stress responses [1]. These disagreements maybe due to the complex properties of NO, whose functions depends on its location and concentration, plant species, as well as the developmental stages of the plant. In addition, most studies examining the function of NO rely on the application of NO donors or scavengers, which may not accurately reflect the function of endogenous NO in plants.

Here, we presented our results showing that salt and drought-treated rice can induce NO accumulation. These results are consistent with reports in many different plants, in which NO accumulation is enhanced under many different stress conditions [37], though several papers indicate that NO content is decreased by iron deficiency in *Zea mays* [43] or by aluminum in *Hibiscus moscheutos* [44]. In addition, under 25 μM arsenate treatment, NO production is induced in *Festuca arundinaceae* [45], but decreased in *Oryza sativa* [46]. Therefore, plant materials with higher or lower endogenous NO levels are needed for further verification of the role of NO in the plant response to environmental stresses. In this study, we further indicated that *nNOS* expression can efficiently increase NOS activity and endogenous NO level in transgenic rice lines, resulting in higher tolerance to both drought and salt stresses.

NO is considered to play a role in stress-induced oxidative damage in plants, though its role in this process is still disputed. Many reports show that exogenous NO application improves abiotic stress tolerance, concomitant with a decrease in H_2_O_2_ and MDA levels [37]. For example, exogenous application of NO inhibits ROS accumulation in many different plants under stress conditions [12,13,40,41]. However, the application of NO inhibitors or scavengers also reduces the stress-caused oxidative damage in several reports [15,16]. Our study revealed that the *nNOS* transgenic rice plants with higher endogenous NO content accumulated less H_2_O_2_ and MDA under drought and high salinity conditions, possibly by up-regulating the expression of *POX* and *CAT* genes.

Proline is also an important regulator for plant tolerance to various stresses such as drought, high salinity, high light and heavy metal stress [47]. Many reports suggest the involvement of NO in changes to proline accumulation in the plant response to different stresses, mainly based on exogenous application of either NO donors or scavengers, but different, even opposite observations have also been reported [29]. For example, exogenously applied NO improves proline content in both wheat and rice under drought stress [48,49], whereas Xiong *et al*. (2012) showed that exogenous NO does not alter proline content in drought-stressed rice and well-watered rice [29]. Our study indicated that the *nNOS* transgenic plants with higher NO content amplified drought/salt-promoted proline accumulation, leading to improved drought stress tolerance.

We noted that a previous work reported that Arabidopsis overexpressing *nNOS* had increased tolerance to both drought and salt stresses [22]. This paper indicated that the transgenic Arabidopsis lines up-regulated the expression of *RD22*, *KIN2* and *COR15A*, of six tested stress-related genes, under normal growth conditions [22]. However, our transgenic rice lines with higher NO levels did not change the expression of all assayed genes under normal growth conditions. Similarly, proline content was elevated in the transgenic Arabidopsis plants, but not in our transgenic rice lines under normal growth conditions. Moreover, our data further indicated that the transgenic rice lines with lower stomatal conductance had higher RWC compared with wild-type rice when subjected to drought and salt stresses. In addition, we further showed in this paper that the transgenic rice plants lowered H_2_O_2_ content by enhancing the expression of antioxidant enzyme genes and their enzymatic activities under stress conditions. This study also provides valuable materials to investigate the role of NO and improve rice, an agronomically important crop for enhanced tolerance to environmental stresses.

In summary, overexpressing rat *nNOS* increased both NOS activity and NO content, resulting in improved drought and salt stress tolerance in rice. Further studies revealed that *nNOS* transgenic plants had stronger water-holding capability, reduced H_2_O_2_ and MDA content, improved proline accumulation and expression of stress-responsive genes under stress conditions for higher tolerance to both drought and salt stresses.

## Supporting Information

S1 Table Primers used for qRT-PCR analysis in this study. F: forward; R: reverse.
(DOC)
