# Supplementary material for: Overexpression of Rat Neurons Nitric Oxide Synthase in Rice Enhances Drought and Salt Tolerance
Source: PLoS One. 2015 Jun 29;10(6):e0131599. doi: 10.1371/journal.pone.0131599 (PMC4485468; doi:10.1371/journal.pone.0131599)
Supplement: S1 Table — F: forward; R: reverse. (DOC) [file pone.0131599.s001.doc]

**S1 Table. Primers used for qRT-PCR analysis in this study.** **F: forward; R: reverse.**

**Primer name Sequence (5’ to 3’)**

*OsDreb2A* F AGATTGCTCCGTGCAAGTG

*OsDreb2A* R CTGGAGCTTCTGGTTTTGCT

*OsDreb2B* F TTTGTGGAAAGGCGACAAG

*OsDreb2B* R TGGATCAACCCCTTCGTAGT

*OsSNAC1* F GTCAAGACTGATTGGATCATGC

*OsSNAC1* R CCAATCATCCAACCTGAGAGA

*OsSNAC2* F TGTGCCGGATTTACAACAAG

*OsSNAC2* R CACCATCGGCTTCCTCTG

*OsLea3* F GGCGCAGTACACCAAGGA

*OsLea3* R ACCTGCTCACTCGCCTGT

*OsRD29A* F TGGATCAAACAGAGGAACCA

*OsRD29A* R CATCTTAGTCGCACCATTCTCA

*OsCATA* F GCCGGATAGACAGGAGAGGT

*OsCATA* R TCTTCACATGCTTGGCTTCA

*OsCATB* F GGTGGGTTGATGCTCTCTCA

*OsCATB* R ATTCCTCCTGGCCGATCTAC

*OsPOX1* F CATCCCAGCTCCCAACAA

*OsPOX1* R AGACATGCCAATGGTGTGG

*nNOS* F CAGAGGAGGACGCTGGTGTA

*nNOS* R CCGCTCGAGATCCAGTTAGGAGCTGAAAAC

*OseEF-1α* F TTTCACTCTTGGTGTGAAGCAGAT

*OseEF-1α* R GACTTCCTTCACGATTTCATCGTAA
